# The Second-Generation PIM Kinase Inhibitor TP-3654 Resensitizes ABCG2-Overexpressing Multidrug-Resistant Cancer Cells to Cytotoxic Anticancer Drugs

**DOI:** 10.3390/ijms22179440

**Published:** 2021-08-30

**Authors:** Chung-Pu Wu, Yan-Qing Li, Ya-Chen Chi, Yang-Hui Huang, Tai-Ho Hung, Yu-Shan Wu

**Affiliations:** 1 Graduate Institute of Biomedical Sciences, College of Medicine, Chang Gung University, Taoyuan 33302, Taiwan; hi9300190@yahoo.com.tw (Y.-Q.L.); ilu171085@gmail.com (Y.-C.C.); yanghui.huang01@gmail.com (Y.-H.H.); 2 Department of Physiology and Pharmacology, College of Medicine, Chang Gung University, Taoyuan 33302, Taiwan; 3 Department of Obstetrics and Gynecology, Taipei Chang Gung Memorial Hospital, Taipei 10507, Taiwan; thh20@adm.cgmh.org.tw; 4 Department of Medicine, College of Medicine, Chang Gung University, Taoyuan 33302, Taiwan; 5 Department of Obstetrics and Gynecology, Keelung Chang Gung Memorial Hospital, Keelung 20401, Taiwan; 6 Department of Chemistry, Tunghai University, Taichung 40704, Taiwan; yushanwu@thu.edu.tw

**Keywords:** multidrug resistance, breast cancer resistance protein, modulator, TP-3654, SGI-9481

## Abstract

Human ATP-binding cassette (ABC) subfamily G member 2 (ABCG2) mediates the transport of a wide variety of conventional cytotoxic anticancer drugs and molecular targeted agents. Consequently, the overexpression of ABCG2 in cancer cells is linked to the development of the multidrug resistance (MDR) phenotype. TP-3654 is an experimental second-generation inhibitor of PIM kinase that is currently under investigation in clinical trials to treat advanced solid tumors and myelofibrosis. In this study, we discovered that by attenuating the drug transport function of ABCG2, TP-3654 resensitizes ABCG2-overexpressing multidrug-resistant cancer cells to cytotoxic ABCG2 substrate drugs topotecan, SN-38 and mitoxantrone. Moreover, our results indicate that ABCG2 does not mediate resistance to TP-3654 and may not play a major role in the induction of resistance to TP-3654 in cancer patients. Taken together, our findings reveal that TP-3654 is a selective, potent modulator of ABCG2 drug efflux function that may offer an additional combination therapy option for the treatment of multidrug-resistant cancers.

## 1. Introduction

The ATP-binding cassette (ABC) proteins ABCB1 (MDR1; P-glycoprotein) and ABCG2 (BCRP; MXR; ABCP) are transmembrane proteins that utilize energy derived from ATP hydrolysis to translocate a wide range of structurally unrelated chemotherapeutic drugs across membranes [1,2,3]. The substrate drugs of ABCB1 and ABCG2 include, but not limited to, some of the most commonly prescribed anticancer drugs, such as anthracyclines, taxanes, *Vinca alkaloids*, mitoxantrone and topotecan, as well as numerous molecular targeted agents [3,4,5,6,7,8,9]. Considering that ABCB1 and ABCG2 overexpression is highly associated with the development of multidrug resistance (MDR) [3,4] and poor prognosis in patients with solid tumors [10] and hematologic malignancies [11,12,13,14,15], discovering therapeutic agents and strategies to overcome the activity of ABCB1 and ABCG2 is of urgent need.

Given the lack of U.S. Food and Drug Administration (FDA)-approved therapeutic agents for the treatment of multidrug-resistant cancer patients, we and others have been exploring the possibility of repositioning FDA-approved drugs or therapeutic agents that have undergone clinical testing for the reversal of MDR mediated by ABCB1 and/or ABCG2 [16,17,18,19,20,21,22,23,24]. TP-3654 (SGI-9481) is a second-generation small-molecule inhibitor of the proviral integration site for Moloney murine leukemia virus (PIM) serine/threonine kinases [25]. The proto-oncogene-encoded PIM kinases (Pim-1, Pim-2, and Pim-3) are known to be involved in tumorigenesis, promoting tumor proliferation and chemo-resistance [26]. More significantly, the overexpression of PIM kinases is associated with poor prognosis in patients with head and neck cancers [27], bladder cancer [28], prostate cancer [29], or hematologic malignancies such as acute myelogenous leukemia (AML) [30], chronic lymphocytic leukemia (CLL) [31], multiple myeloma (MM) and B-cell lymphoma [32]. Consequently, PIM kinases have been considered as attractive molecular targets for cancer chemotherapy [33,34,35]. The tolerability, pharmacokinetics and pharmacodynamics, as well as antitumor activity of TP-3654, are currently being evaluated in patients with advanced solid tumors (ClinicalTrials.gov Identifier: NCT03715504) and patients with myelofibrosis (NCT04176198).

Providing that numerous protein kinase inhibitors [22,36,37,38,39,40], including PIM inhibitor [41], were reported to interact with ABCB1 and ABCG2, we investigated the effect of TP-3654 on ABCB1- and ABCG2-mediated drug transport and multidrug resistance in a panel of drug-sensitive cancer cell lines and their respective ABCB1- and ABCG2-overexpressing multidrug-resistant cancer cell lines. We found that ABCB1 and ABCG2 did not confer resistance to TP-3654. More significantly, TP-3654 selectively interferes with drug transport mediated by ABCG2 and resensitizes ABCG2-overexpressing cancer cells to cytotoxic anticancer drugs. Collectively, these findings reveal an additional pharmacological activity of TP-3654 that reverses ABCG2-mediated MDR and may be an effective therapeutic strategy for patients with multidrug-resistant cancers.

## 2. Results

### 2.1. TP-3654 Is Equally Cytotoxic to ABCB1- and ABCG2-Overexpressing Multidrug-Resistant Cancer Cells as to Drug-Sensitive Cancer Cells

To determine whether cells overexpressing ABCB1 or ABCG2 are less susceptible to TP-3654 treatment, we determined the cytotoxicity of TP-3654 in human KB-3-1 epidermal cancer cell line, human OVCAR-8 ovarian cancer cell line and their respective ABCB1-overexpressing variants KB-V-1 and NCI-ADR-RES, human S1 colon cancer cell line, human H460 non-small cell lung cancer (NSCLC) cell line and their respective ABCG2-overexpressing variants S1-M1-80 and H460-MX20, as well as in pcDNA3.1-HEK293 (HEK293 stably transfected with empty pcDNA 3.1 vector), MDR19-HEK293 (HEK293 stably transfected with human ABCB1), and R482-HEK293 (HEK293 stably transfected with human ABCG2) cells. As summarized in Table 1, drug-sensitive parental cells are equally sensitive to TP-3654 as ABCB1- and ABCG2-overexpressing cells, with IC_50_ values ranging from approximately 3 to 35 μM.

### 2.2. TP-3654 Selectively Reverses Multidrug Resistance in ABCG2-Overexpressing Cancer Cells

**Figure 1:**
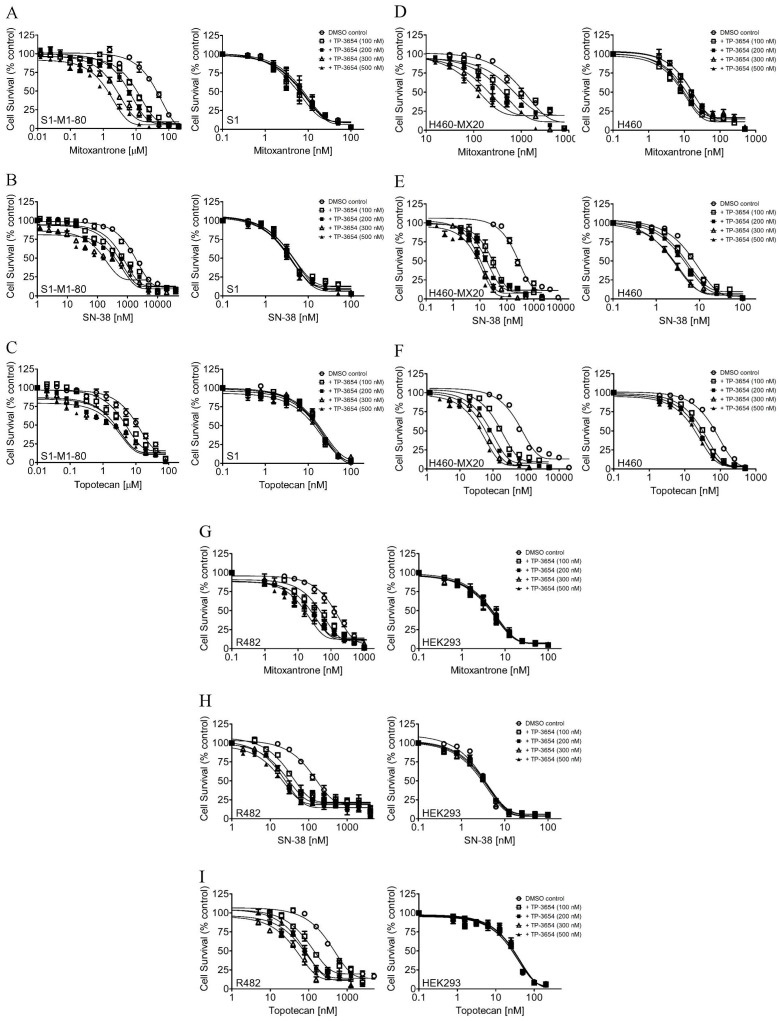
TP-3654 resensitizes ABCG2-overexpressing cancer cells to cytotoxic anticancer drugs. The cytotoxicity of mitoxantrone, SN-38, and topotecan was determined in ABCG2-overexpressing S1-M1-80 (**A**–**C**, left panels) and drug-sensitive parental S1 (**A**–**C**, right panels) cancer cells, ABCG2-overexpressing H460-MX20 (**D**–**F**, left panels), and drug-sensitive H460 (**D**–**F**, right panels) cancer cells, as well as ABCG2-transfected R482-HEK293 (**G**–**I**, left panels) and parental HEK293 (**G**–**I**, right panels) cells in the presence of DMSO (open circles) or TP-3654 at 100 nM (open squares), 200 nM (filled squares), 300 nM (open triangles), or 500 nM (filled triangles). Data represent means ± S.E.M. from at least three independent experiments.

To determine the selectivity and potency of TP-3654 on reversing transporter-mediated MDR, we investigated the chemosensitizing effect of TP-3654 on MDR mediated by ABCB1 or ABCG2 in ABCB1- and ABCG2-overexpressing multidrug-resistant cells. We first examined the effect of TP-3654 on ABCB1-mediated resistance to doxorubicin, paclitaxel and colchicine, three well-known substrate drugs of ABCB1 [42], in ABCB1-overexpressing NCI-ADR-RES, KB-V-1 cancer cells, and ABCB1-transfected MDR19-HEK293 cells. As summarized in Table 2, we found that at submicromolar concentrations (100–500 nM), TP-3654 had only a marginal effect on ABCB1-mediated MDR in all three ABCB1-overexpressing cell lines. In contrast, we discovered that TP-3654 significantly resensitized ABCG2-overexpressing S1-M1-80 (Figure 1A–C), H460-MX20 (Figure 1D–F) cancer cells and ABCG2-transfected R482-HEK293 cells (Figure 1G–I) to ABCG2 substrate drugs mitoxantrone, SN-38 and topotecan [43,44], in a concentration-dependent manner. The IC_50_ values and extent of reversal by TP-3654, represented by the fold-reversal (FR) values [16,45], are summarized in Table 2 and Table 3. Of note, since a moderate basal level of ABCG2 is expressed in H460 cells [46], we also detected some MDR reversal effects by TP-3654 in H460 cells. Our results revealed that at submicromolar concentrations, TP-3654 selectively reversed ABCG2-mediated MDR in ABCG2-overexpressing multidrug-resistant cancer cells in a concentration-dependent manner.

### 2.3. TP-3654 Selectively Inhibits the Drug Efflux Function of ABCG2

**Figure 2:**
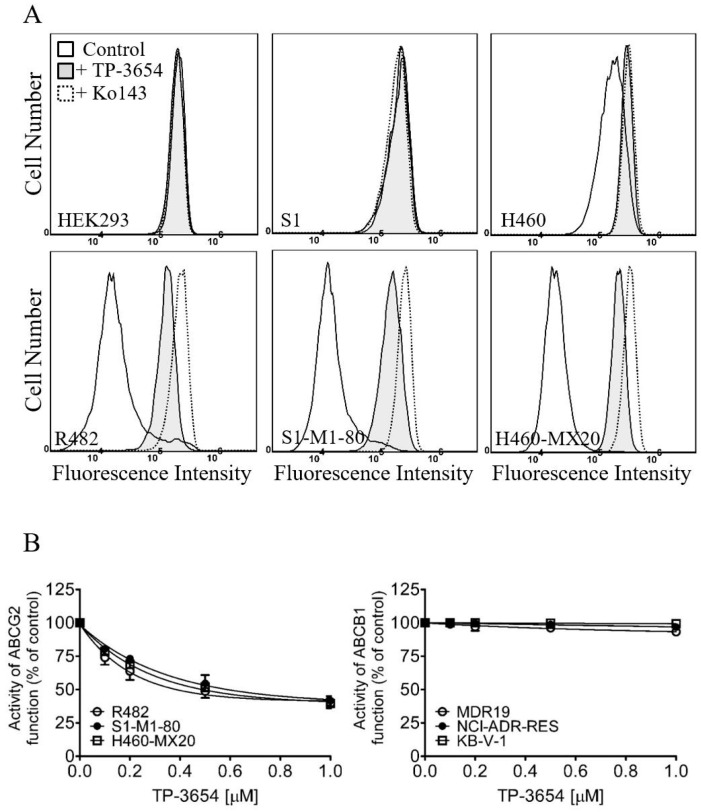
TP-3654 increases the intracellular accumulation of pheophorbide A (PhA) in ABCG2-overexpressing cells. (**A**) Flow cytometry analysis of PhA accumulation in parental HEK293 cells and ABCG2-transfected R482-HEK293 cells (left panels), parental S1 and ABCG2-overexpressing S1-M1-80 cells (middle panels), and parental H460 and ABCG2-overexpressing H460-MX20 cells (right panels) treated with DMSO (control, solid lines), 1 μM of TP-3654 (filled solid lines), or 1 μM of Ko143 as a positive control (dotted lines) for ABCG2. (**B**) Effect of increasing concentrations (0–1 μM) of TP-3654 on ABCG2-mediated efflux of PhA (left panel) in ABCG2-overexpressing R482-HEK293 cells (open circles), S1-M1-80 (filled circles), and H460-MX20 cells (open squares), and on ABCB1-mediated efflux of calcein-AM (right panel) in ABCB1-overexpressing MDR19-HEK293 cells (open circles), NCI-ADR-RES (filled circles), and KB-V-1 cells (open squares). Data represent means ± S.D. from at least three independent experiments.

The effect of TP-3654 on the drug efflux function of ABCB1 and ABCG2 was determined by treating ABCB1-overexpressing cells and ABCG2-overexpressing cells, respectively, with calcein-AM or pheophorbide A (PhA), in the absence or presence of TP-3654 as described in Section 4. As shown in Figure 2, increasing concentrations (0–1 μM) of TP-3654 had no significant effect on ABCB1-mediated efflux of calcein in ABCB1-transfected MDR19-HEK293 cells (open circles), ABCB1-overexpressing NCI-ADR-RES (filled circles), or KB-V-1 cancer cells (open squares). In contrast, TP-3654 inhibited ABCG2-mediated efflux of PhA in ABCG2-transfected R482-HEK293 cells (open circles), ABCG2-overexpressing S1-M1-80 (filled circles), and H460-MX20 cancer cells (open squares) in a concentration-dependent manner (Figure 2B), with approximately 60% of the maximum inhibition by 3 μM of Ko143 and the IC_50_ values of approximately 140, 240, and 200 nM, respectively.

### 2.4. TP-3654 Enhances Drug-Induced Apoptosis in ABCG2-Overexpressing Cancer Cells

**Figure 3:**
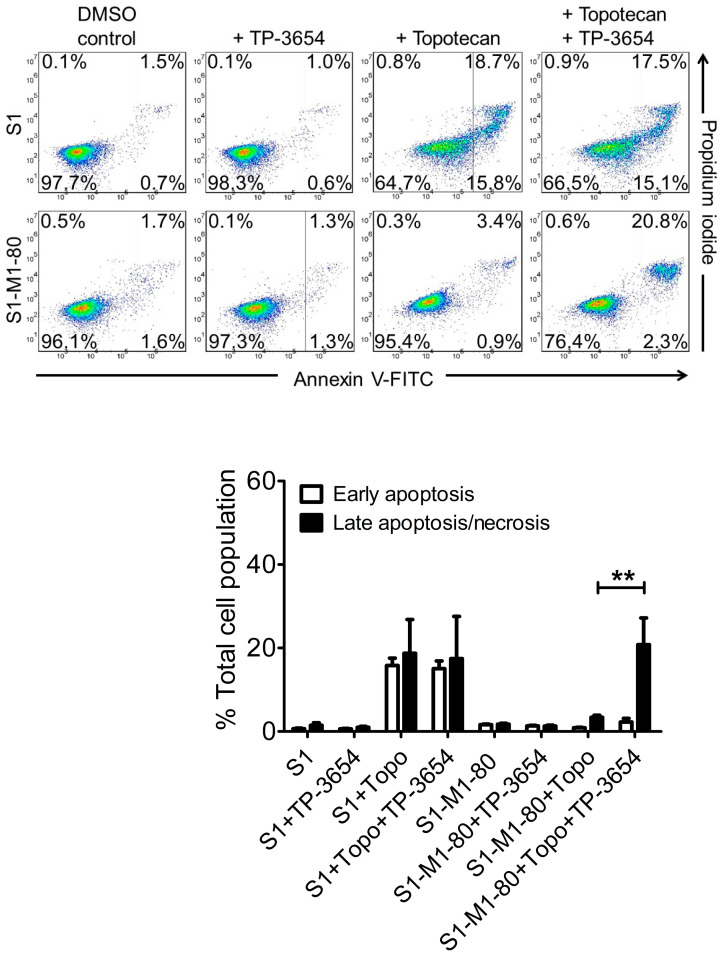
TP-3654 improves drug-induced apoptosis in ABCG2-overexpressing cancer cells. The extent of apoptosis was determined by treating drug-sensitive S1 and ABCG2-overexpressing multidrug-resistant S1-M1-80 cancer cells with DMSO (control), 500 nM of TP-3654 (+TP-3654), 5 μM of topotecan (+topotecan), or a combination of 5 μM of topotecan with 500 nM of TP-3654 (+topotecan +TP-3654) and analyzed by flow cytometry as described in Section 4. The quantification results are presented as mean ± S.D. calculated from at least three independent experiments. ** *p* < 0.01, versus the same treatment in the absence of TP-3654.

Next, knowing that TP-3654 selectively reverses ABCG2-mediated MDR (Figure 1) by inhibiting the drug transport function of ABCG2 (Figure 2), we examined the effect of TP-3654 on drug-induced apoptosis in ABCG2-overexpressing cancer cells. Drug-sensitive parental S1 and ABCG2-overexpressing multidrug-resistant S1-M1-80 cancer cells were treated with DMSO (control), 0.5 μM of TP-3654, 5 μM of topotecan, or a combination of 5 μM of topotecan with 0.5 μM of TP-3654 for 48 h before processed as detailed in Section 4. As shown in Figure 3, TP-3654 had no significant apoptotic effect on S1 or S1-M1-80 cancer cells, whereas a substantial increase of apoptosis, from approximately 2% basal to 35% total apoptosis, was observed in S1 cancer cells. Notably, we discovered that TP-3654 enhanced topotecan-induced apoptosis, from approximately 4% to 23% total apoptosis, in S1-M1-80 cancer cells. Our results here confirmed that TP-3654 resensitized ABCG2-overexpressing cancer cells to ABCG2 substrate drug by enhancing drug-induced apoptosis, and not by initiating growth retardation.

### 2.5. TP-3654 Treatment Does Not Affect the Protein Expression of ABCG2 in ABCG2-Overexpressing Cancer Cells

**Figure 4:**
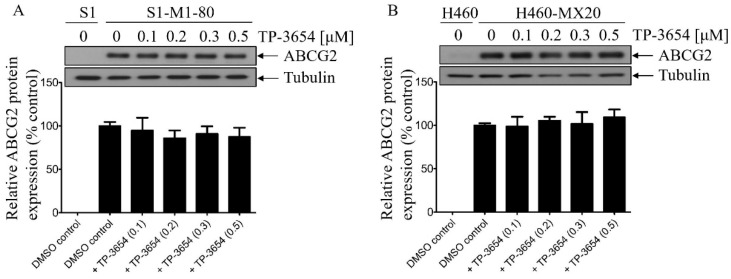
TP-3654 does not alter the protein expression of ABCG2 in ABCG2-overexpressing cancer cells. (**A**) S1-M1-80 and (**B**) H460-MX20 cancer cells were treated with DMSO (control) or increasing concentrations (0–0.5 μM) of TP-3654 for 72 h and processed for Western blotting as described in Section 4. The representative immunoblots (upper panel) and the corresponding quantification (lower panel) of human ABCG2 protein are shown. α-Tubulin was used as an internal loading control. Data represent means ± S.D. from at least three independent experiments.

Natarajan et al. reported previously that the PIM kinase inhibitor SG-1776 reversed ABCG2-mediated MDR and increased apoptosis of cells overexpressing ABCG2 by decreasing the protein expression of ABCG2 [41]. To this end, we examined the effect of TP-3654 on the protein expression of ABCG2 in ABCG2-overexpressing cancer cells (Figure 4 and Figure S1). ABCG2-overexpressing S1-M1-80 and H460-MX20 cancer cells were treated with DMSO (control) or increasing concentrations (0.1–0.5 μM) of TP-3654 for 72 h followed by Western blot analysis as described in Section 4. We discovered that TP-3654 did not significantly affect the protein expression of ABCG2 in S1-M1-80 (Figure 4A) or H460-MX20 (Figure 4B) cancer cells. Our data indicated that TP-3654 reverses ABCG2-mediated MDR and increased apoptosis of ABCG2-overexpressing cancer cells by inhibiting the drug transport function of ABCG2.

### 2.6. Docking of TP-3654 in the Drug-Binding Pocket of ABCG2

**Figure 5:**
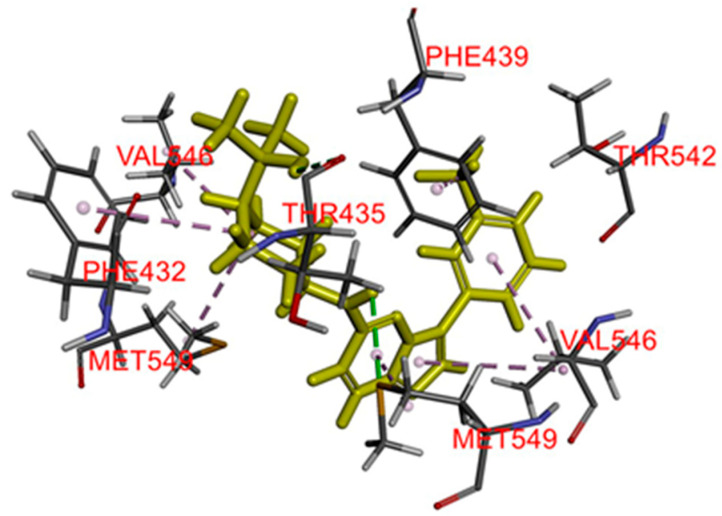
The binding mode of TP-3654 with ABCG2 protein structure (PDB:6VXH) was predicted by Accelrys Discovery Studio 4.0 software as described in Section 4. TP-3654 is shown as a molecular model with highlighted yellow color and the atoms for interacting amino acid residues were colored as carbon (gray), oxygen (red), hydrogen (light gray), and nitrogen (blue). Dotted lines indicate proposed interactions.

To further understand the binding interaction of TP-3654 with ABCG2, a binding study was performed. TP-3654 was docked into the substrate-binding cavity between the transmembrane helices of the human ABCG2 structure (PDB:6VXH), and the best binding conformation was selected with the binding energy calculated to be −58.23 kcal/mol. Hydrophobic interactions were observed between TP-3654 with both monomers A and B. Val^546^, Met^549^ on monomer A and Phe^432^ on monomer B were predicted to interact with the cyclohexane moiety on TP-3654. Met^549^ and Val^546^ on monomer B were found to interact with the imidazo [1,2-b]pyridazine ring. More interactions were also predicted between Val^546^ on monomer B with the phenyl ring and Phe^439^ with CF_3_. One hydrogen bond was predicted between Thr^435^ on monomer B and the hydroxyl group on TP-3654 (Figure 5).

## 3. Discussion

Preclinical development of synthetic inhibitors of ABCB1 and/or ABCG2 has not been successful due to the lack of selectivity and unexpected adverse drug–drug interactions [3,47,48,49,50]. In recent years, many protein kinase inhibitors were found to interact with ABCB1 and/or ABCG2. Some kinase inhibitors such as almonertinib [24], sitravatinib [51], erdafitinib [23], avapritinib [45], and midostaurin [52], inhibit drug efflux mediated by ABCB1 and/or ABCG2, whereas some kinase inhibitors such as osimertinib [53], encorafenib [54], ibrutinib [55], and vemurafenib [56], are substrates of ABCB1 and/or ABCG2. More importantly, the results of combination therapy trials of erlotinib and gemcitabine for advanced pancreatic cancer patients [57,58], as well as lapatinib and capecitabine for human epidermal growth factor receptor 2 (HER2)-positive advanced breast cancer patients [59,60] demonstrated the advantages of combination therapy of kinase inhibitors with conventional chemotherapeutic drugs over monotherapy. Furthermore, findings from a more recent trial of doxorubicin in combination with the ABCB1-modulating nilotinib showed the benefits of including a kinase inhibitor in combination therapy against multidrug-resistant cancers [61]. These findings prompted us to investigate the interactions between TP-3654 and ABCB1 and ABCG2.

In this study, we discovered that the PIM kinase inhibitor TP-3654 could inhibit ABCG2-mediated drug transport in a concentration-dependent manner. Consequently, the extent of drug-induced apoptosis and MDR mediated by ABCG2 were significantly reversed by TP-3654 in ABCG2-overexpressing multidrug-resistant cells. We further demonstrated that TP-3654 has no significant effect on ABCG2 protein expression in ABCG2-overexpressing cancer cells. In contrast, TP-3654 had a minimal effect on the transport function of ABCB1, and it did not resensitize ABCB1-overexpressing cells to ABCB1 substrate drugs. Our results indicate that TP-3654 is selective to ABCG2 relative to ABCB1. The in silico molecular docking analysis of TP-3654 in the inward-open conformation of human ABCG2 shows the predicted interactions between TP-3654 and several residues within the substrate-binding pocket of ABCG2. Moreover, despite the interaction with ABCG2, we found that TP-3654 is equally cytotoxic to ABCG2-overexpressing cell lines as to their respective drug-sensitive parental cell lines. Our data suggest that ABCG2 does not confer significant resistance to TP-3654 and may not play a major role in the induction of resistance to TP-3654 in cancer patients. Notably, Natarajan et al. reported that the first clinically tested PIM inhibitor SGI-1776 [62] increased substrate drug-induced apoptosis in ABCB1- and ABCG2-overexpressing multidrug-resistant cancer cells. Moreover, at a non-cytotoxic concentration of 1 μM, SGI-1776 resensitized ABCB1-overexpressing cancer cells to ABCB1 substrate drug daunorubicin with FR values of 2.9 and 4.0, whereas it resensitized ABCG2-overexpressing cancer cells to ABCG2 substrate drug mitoxantrone with FR values of 2.7 and 2.4 [41]. In contrast, at a non-cytotoxic concentration of 500 nM, TP-3654 did not resensitize ABCB1-overexpressing cancer cells to ABCB1 substrate drugs, but significantly resensitized ABCG2-overexpressing cancer cells to ABCG2 substrate drugs topotecan, SN-38, and mitoxantrone, with FR values ranging from approximately 4 to 32 (Table 3). Our results suggest that TP-3654 is more potent and selective than SGI-1776 against ABCG2-mediated MDR.

## 4. Materials and Methods

### 4.1. Chemicals

Fluorescein isothiocyanate (FITC) Annexin V Apoptosis Detection Kit was purchased from BD Pharmingen (San Diego, CA, USA). Tools Cell Counting (CCK-8) kit was acquired from Biotools Co., Ltd. (Taipei, Taiwan). TP-3654 was obtained from Selleckchem (Houston, TX, USA). ABCB1 reference inhibitor tariquidar, ABCG2 reference inhibitor Ko143, and all other chemicals were purchased from Sigma-Aldrich (St. Louis, MO, USA) unless stated otherwise.

### 4.2. Cell Lines

Parental human epidermal cancer cell line KB-3-1 and its ABCB1-overexpressing variant cell line KB-V-1 [63]; the human embryonic kidney HEK293 cell line stably transfected with either empty pcDNA 3.1 vector, ABCB1-transfected MDR19-HEK293 cell line [64] and ABCG2-transfected R482-HEK293 cell line [65] were cultured in Dulbecco’s modified Eagle’s medium (DMEM) (Gibco, Invitrogen, Carlsbad, CA, USA). NCI-ADR-RES cell line was maintained in the presence of 0.85 μM doxorubicin [66], whereas KB-V-1 cell line was maintained in the presence of 1 mg/mL vinblastine [67]. Parental human colon cancer cell line S1 and its ABCG2-overexpressing variant cell line S1-M1-80; parental human ovarian cancer cell line OVCAR-8 and its ABCB1-overexpressing variant cell line NCI-ADR-RES [66]; parental human NSCLC cell line H460 and its ABCG2-overexpressing variant cell line H460-MX20 [68] were cultured in Rosewell Park Memorial Institute (RPMI) 1640 (Gibco, Invitrogen, Carlsbad, CA, USA). S1-M1-80 cell line was maintained in the presence of 80 nM mitoxantrone [69], whereas the H460-MX20 cell line was maintained in the presence of 20 nM of mitoxantrone [70]. Cell lines were maintained in medium supplemented with 10% fetal calf serum (FCS), 2 mM l-glutamine, 2 mg/mL G418, and 100 units of penicillin/streptomycin/mL at 37 °C in 5% CO_2_ humidified air. Cell lines were generous gifts from Dr. Michael M. Gottesman and Dr. Susan E. Bates, National Cancer Institute, Bethesda, MD, USA.

### 4.3. Cytotoxicity Assay

Cells were seeded in 96-well flat-bottom plates and allowed to attach overnight at 37 °C in 5% CO_2_ humidified air. Varying concentrations of TP-3654 alone or in combination with chemotherapeutic agents were added to each plate and incubated for an additional 72 h before processed as previously described [71]. Viable cells were quantified based on the cytotoxic MTT assay reported by Ishiyama et al. [72]. IC_50_ values were calculated using the fitted concentration–response curve of each drug regimen from at least three independent experiments. The extent of reversal was presented by a fold-reversal (FR) value, determined by adding TP-3654 or tariquidar or Ko143 to the cytotoxicity assays as described previously [16].

### 4.4. Apoptosis Assay

The concurrent staining of annexin V–FITC and propidium iodide (PI) method was used according to the manufacturer’s instructions (BD Pharmingen) to determine the extent of apoptosis induced by a cytotoxic drug and as previously described [51]. Briefly, S1 and S1-M1-80 cancer cells were treated with DMSO, 500 nM of TP-3654 alone, 5 µM of topotecan alone, or the combination of 5 µM of topotecan and 500 nM of TP-3654 as indicated for 48 h before stained with annexin V–FITC (1.25 µg/mL) and PI (0.1 mg/mL) for 15 min at room temperature. Samples were analyzed by FACScan equipped with the CellQuest software (Becton-Dickinson Biosciences, San Jose, CA, USA) as previously described [19].

### 4.5. Flow Cytometry

Flow cytometry assays with the ABCB1 substrate calcein-AM and the ABCG2 substrate PhA were performed as described previously [51]. Briefly, trypsinized cells were incubated in phenol red-free Iscove’s modified Dulbecco’s medium (IMDM) supplemented with 10% FCS and 100 units of penicillin/streptomycin/mL with calcein-AM or PhA in the presence of DMSO (control) or 3 μM of tariquidar or 1 μM of Ko143 or increasing concentrations of TP-3654. The relative fluorescence intensity was detected using a FACSort flow cytometer (Becton-Dickinson) and analyzed using FlowJo software (Tree Star, Inc., Ashland, OR, USA), as described previously [38,73].

### 4.6. Immunoblot

An immunoblot assay for ABCG2 was performed on ABCG2-overexpressing S1-M1-80 and H460-MX20 cancer cells using the BXP-21 (1:15000 dilution) antibody (Abcam, Cambridge, MA, USA), as previously described [8]. The α-tubulin (1:100,000 dilution) antibody (Sigma-Aldrich, St. Louis, MO, USA) was used to detect the positive loading control tubulin. The horseradish peroxidase-conjugated goat anti-mouse immunoglobulin G (IgG) (1:100,000 dilution) (Sigma-Aldrich, St. Louis, MO, USA) was used as the secondary antibody. Signals were detected using the enhanced chemiluminescence (ECL) kit (Merck Millipore, Billerica, MA, USA).

### 4.7. Docking Analysis of TP-3654 with ABCG2

The structures of ABCG2 protein (PDB:6VXH) [74] and TP-3654 were first prepared with CHARMM force field at pH 7.4 using Accelrys Discovery Studio 4.0. Docking of TP-3654 in ABCG2 was performed using CDOCKER module of the same software. The docked poses with the lowest CDOCKER interaction energy were selected and the respective interaction energy was calculated as described previously [9].

### 4.8. Quantification and Statistical Analysis

Experimental results were obtained from at least three independent experiments. The differences were analyzed by a two-tailed Student’s t-test and labeled with asterisks as “statistically significant” if the probability, *p*, was less than 0.05 compared with control. GraphPad Prism software (GraphPad Software, La Jolla, CA, USA) was used for curve plotting, and KaleidaGraph software (Synergy Software, Reading, PA, USA) was used for statistical analysis.

## 5. Conclusions

**Figure 6:**
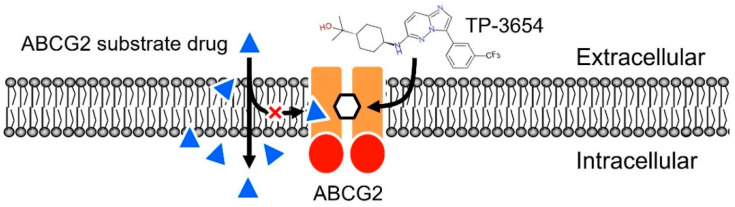
Schematic diagram showing TP-3654 resensitizing ABCG2-overexpressing multidrug-resistant cancer cells to cytotoxic anticancer drugs. By outcompeting the binding of ABCG2 substrate drugs (blue triangles), TP-3654 inhibits the drug efflux function of ABCG2 and increases the efficacy of ABCG2 substrate drugs in ABCG2-overexpressing cancer cells.

In summary, we revealed that the second-generation PIM kinase inhibitor TP-3654 resensitizes ABCG2-overexpressing multidrug-resistant cancer cells to cytotoxic anticancer drugs by attenuating the drug efflux function of ABCG2 (Figure 6). Although it is possible that unforeseen drug–drug interactions and adverse drug reactions may occur, our results warrant further investigation in the combination therapy of TP-3654 and cytotoxic substrate drugs of ABCG2 against multidrug-resistant tumors.

## Figures and Tables

**Table 1:** Sensitivity of drug-sensitive and multidrug-resistant cells overexpressing ABCB1 or ABCG2 to TP-3654.

Cell Line	Type	Transporter Expressed	IC_50_ (μM) ^†^
KB-3-1	Epidermal	-	19.07 ± 1.59
KB-V-1	Epidermal	ABCB1	24.50 ± 3.24
OVCAR-8	Ovarian	-	34.33 ± 5.92
NCI-ADR-RES	Ovarian	ABCB1	28.00 ± 3.38
S1	Colon	-	4.10 ± 1.17
S1-M1-80	Colon	ABCG2	3.39 ± 0.39
H460	Lung	-	22.26 ± 3.35
H460-MX20	Lung	ABCG2	22.05 ± 3.87
pcDNA3.1-HEK293	-	-	2.76 ± 0.79
MDR19-HEK293	-	ABCB1	3.03 ± 0.59
R482-HEK293	-	ABCG2	2.55 ± 0.53

^†^ IC_50_ values are mean ± SD calculated from dose–response curves obtained from at least three independent experiments using cytotoxicity assay as described in Section 4.

**Table 2:** Chemosensitizing effect of TP-3654 on ABCB1-mediated multidrug resistance in ABCB1-overexpressing human cell lines.

Treatment	Concentration (nM)	Mean IC_50_^†^ ± SD and (FR ^‡^)	
		OVCAR-8 (Parental) (nM)	NCI-ADR-RES (Resistant) (μM)
Paclitaxel	-	3.82 ± 0.62 (1.0)	9.37 ± 1.47 (1.0)
+TP-3654	100	4.47 ± 0.65 (0.9)	10.12 ± 1.20 (0.9)
+TP-3654	200	4.18 ± 0.61 (0.9)	9.34 ± 1.24 (1.0)
+TP-3654	300	4.46 ± 0.66 (0.9)	9.17 ± 1.15 (1.0)
+TP-3654	500	4.12 ± 0.47 (0.9)	7.36 ± 0.84 (1.3)
+tariquidar	1000	3.56 ± 0.59 (1.1)	12.74 ± 1.32 (nM) *** (735)
		**(nM)**	**(μM)**
Doxorubicin	-	248.55 ± 52.08 (1.0)	6.30 ± 0.67 (1.0)
+TP-3654	100	242.57 ± 41.55 (1.0)	7.26 ± 1.11 (0.9)
+TP-3654	200	226.91 ± 51.04 (1.1)	8.35 ± 1.15 (0.8)
+TP-3654	300	190.94 ± 35.52 (1.3)	7.63 ± 1.12 (0.8)
+TP-3654	500	201.04 ± 48.62 (1.2)	6.86 ± 0.87 (0.9)
+tariquidar	1000	216.42 ± 50.37 (1.1)	0.36 ± 0.07 *** (17.5)
		**(nM)**	**(nM)**
Colchicine	-	33.25 ± 11.26 (1.0)	1827.27 ± 229.42 (1.0)
+TP-3654	100	34.30 ± 13.08 (1.0)	2300.41 ± 525.88 (0.8)
+TP-3654	200	35.92 ± 13.87 (0.9)	2185.00 ± 387.82 (0.8)
+TP-3654	300	35.99 ± 12.62 (0.9)	2135.46 ± 235.40 (0.9)
+TP-3654	500	32.94 ± 12.25 (1.0)	2672.43 ± 581.86 (0.7)
+tariquidar	1000	34.98 ± 12.35 (0.9)	65.29 ± 23.07 *** (28.0)
**Treatment**	**Concentration** **(nM)**	**KB-3-1 (Parental)** **(nM)**	**KB-V-1 (Resistant)** **(nM)**
Paclitaxel	-	2.31 ± 0.75 (1.0)	2083.57 ± 250.53 (1.0)
+TP-3654	100	2.46 ± 0.64 (0.9)	2269.00 ± 245.85 (0.9)
+TP-3654	200	2.26 ± 0.52 (1.0)	2171.55 ± 290.13 (1.0)
+TP-3654	300	2.07 ± 0.48 (1.1)	2263.61 ± 271.75 (0.9)
+TP-3654	500	2.15 ± 0.42 (1.1)	1648.56 ± 187.39 (1.3)
+tariquidar	1000	2.24 ± 0.59 (1.0)	2.16 ± 0.59 *** (964.6)
		**(nM)**	**(μM)**
Doxorubicin	-	174.34 ± 41.49 (1.0)	2.27 ± 0.21 (1.0)
+TP-3654	100	153.79 ± 43.44 (1.1)	2.66 ± 0.21 (0.9)
+TP-3654	200	161.07 ± 44.68 (1.1)	2.33 ± 0.20 (1.0)
+TP-3654	300	209.34 ± 59.06 (0.8)	1.85 ± 0.19 (1.2)
+TP-3654	500	153.50 ± 43.54 (1.1)	1.95 ± 0.18 (1.1)
+tariquidar	1000	185.90 ± 73.46 (0.9)	79.29 ± 23.05 (nM) *** (28.6)
		**(nM)**	**(nM)**
Colchicine	-	20.59 ± 8.45 (1.0)	854.44 ± 71.42 (1.0)
+TP-3654	100	20.55 ± 8.10 (1.0)	921.75 ± 113.96 (0.9)
+TP-3654	200	20.98 ± 7.97 (1.0)	808.36 ± 79.91 (1.1)
+TP-3654	300	19.43 ± 7.17 (1.1)	947.28 ± 98.51 (0.9)
+TP-3654	500	20.56 ± 7.12 (1.0)	914.81 ± 109.63 (0.9)
+tariquidar	1000	19.00 ± 7.23 (1.1)	20.36 ± 6.30 *** (42.0)
**Treatment**	**Concentration** **(nM)**	**pcDNA3.1-HEK293 (Parental) (nM)**	**MDR19-HEK293** **(Resistant) (nM)**
Paclitaxel	-	3.47 ± 0.67 (1.0)	737.62 ± 126.44 (1.0)
+TP-3654	100	4.11 ± 0.97 (0.8)	743.54 ± 146.73 (1.0)
+TP-3654	200	4.02 ± 0.74 (0.9)	581.71 ± 90.37 (1.3)
+TP-3654	300	4.14 ± 0.75 (0.8)	738.96 ± 108.01 (1.0)
+TP-3654	500	3.68 ± 0.63 (0.9)	642.38 ± 132.50 (1.1)
+tariquidar	1000	2.74 ± 0.43 (1.3)	2.05 ± 0.39 *** (359.8)
		**(nM)**	**(nM)**
Doxorubicin	-	25.55 ± 6.31 (1.0)	297.78 ± 51.34 (1.0)
+TP-3654	100	28.60 ± 7.10 (0.9)	294.19 ± 52.47 (1.0)
+TP-3654	200	28.77 ± 7.62 (0.9)	237.93 ± 46.90 (1.3)
+TP-3654	300	27.38 ± 7.99 (0.9)	273.57 ± 78.20 (1.1)
+TP-3654	500	28.61 ± 9.59 (0.9)	272.00 ± 69.14 (1.1)
+tariquidar	1000	22.26 ± 5.37 (1.1)	16.59 ± 2.33 *** (18.0)
		**(nM)**	**(nM)**
Colchicine	-	13.02 ± 2.79 (1.0)	100.07 ± 21.01 (1.0)
+TP-3654	100	15.98 ± 4.05 (0.8)	107.28 ± 21.50 (0.9)
+TP-3654	200	14.95 ± 3.73 (0.9)	78.54 ± 15.95 (1.3)
+TP-3654	300	14.11 ± 3.10 (0.9)	98.65 ± 20.29 (1.0)
+TP-3654	500	14.91 ± 3.50 (0.9)	89.50 ± 22.14 (1.1)
+tariquidar	1000	14.12 ± 3.29 (0.9)	8.46 ± 2.66 ** (11.8)

Abbreviation: FR, fold-reversal. ^†^ IC_50_ values are mean ± SD calculated from dose–response curves obtained from at least three independent experiments using cytotoxicity assay as described in Section 4. ^‡^ FR values were calculated by dividing IC_50_ values of cells treated with a particular substrate drug by IC_50_ values of cells treated with the same substrate drug in the presence of TP-3654 or tariquidar. ** *p* < 0.01; *** *p* < 0.001.

**Table 3:** Chemosensitizing effect of TP-3654 on ABCG2-mediated multidrug resistance in ABCG2-overexpressing human cell lines.

Treatment	Concentration (nM)	Mean IC_50_^†^ ± SD and (FR ^‡^)	
		S1 (Parental) (nM)	S1-M1-80 (Resistant) (μM)
Topotecan	-	12.82 ± 3.16 (1.0)	10.81 ± 0.91 (1.0)
+TP-3654	100	14.43 ± 3.06 (0.9)	5.09 ± 0.76 ** (2.1)
+TP-3654	200	13.66 ± 3.22 (0.9)	2.90 ± 0.64 *** (3.7)
+TP-3654	300	15.93 ± 3.49 (0.8)	2.94 ± 0.70 *** (3.7)
+TP-3654	500	12.94 ± 2.63 (1.0)	2.75 ± 0.73 *** (3.9)
+Ko143	1000	13.23 ± 2.63 (1.0)	1.13 ± 0.24 *** (9.6)
		**(nM)**	**(nM)**
SN-38	-	3.22 ± 0.54 (1.0)	1804.90 ± 112.67 (1.0)
+TP-3654	100	4.14 ± 0.74 (0.8)	706.84 ± 106.11 *** (2.6)
+TP-3654	200	3.32 ± 0.54 (1.0)	517.32 ± 100.26 *** (3.5)
+TP-3654	300	3.67 ± 0.64 (0.9)	443.73 ± 101.80 *** (4.1)
+TP-3654	500	3.14 ± 0.62 (1.0)	304.09 ± 81.75 *** (5.9)
+Ko143	1000	2.69 ± 0.47 (1.2)	104.36 ± 22.88 *** (17.3)
		**(nM)**	**(μM)**
Mitoxantrone	-	5.65 ± 0.48 (1.0)	35.04 ± 6.71 (1.0)
+TP-3654	100	5.10 ± 0.34 (1.1)	10.50 ± 1.03 ** (3.3)
+TP-3654	200	5.94 ± 0.42 (1.0)	5.42 ± 0.57 ** (6.5)
+TP-3654	300	5.92 ± 0.36 (1.0)	2.53 ± 0.25 ** (13.8)
+TP-3654	500	6.22 ± 0.44 (0.9)	1.30 ± 0.11 *** (27.0)
+Ko143	1000	5.34 ± 0.61 (1.1)	0.36 ± 0.04 *** (58.4)
**Treatment**	**Concentration** **(nM)**	**H460 (Parental)** **(nM)**	**H460-MX20 (Resistant) (nM)**
Topotecan	-	60.21 ± 10.05 (1.0)	737.60 ± 145.43 (1.0)
+TP-3654	100	26.93 ± 4.35 ** (2.2)	161.36 ± 26.08 ** (4.6)
+TP-3654	200	20.54 ± 3.24** (2.9)	76.96 ± 12.29 ** (9.6)
+TP-3654	300	21.23 ± 2.99 ** (2.8)	51.10 ± 10.63 ** (14.4)
+TP-3654	500	16.81 ± 2.34 ** (3.6)	37.09 ± 6.65 ** (19.9)
+Ko143	1000	21.53 ± 3.25 ** (2.8)	25.10 ± 5.50 ** (29.4)
		**(nM)**	**(nM)**
SN-38	-	6.10 ± 1.21 (1.0)	246.21 ± 44.64 (1.0)
+TP-3654	100	4.29 ± 0.66 (1.4)	27.87 ± 2.58 ** (8.8)
+TP-3654	200	3.61 ± 0.33 * (1.7)	18.00 ± 2.98 *** (13.7)
+TP-3654	300	1.98 ± 0.22 ** (3.1)	9.20 ± 1.33 *** (26.8)
+TP-3654	500	2.05 ± 0.19 ** (3.0)	7.72 ± 1.92 *** (31.9)
+Ko143	1000	2.10 ± 0.27 ** (2.9)	2.57 ± 0.66 *** (95.8)
		**(nM)**	**(nM)**
Mitoxantrone	-	15.42 ± 1.83 (1.0)	996.71 ± 127.75 (1.0)
+TP-3654	100	8.83 ± 0.99 ** (1.7)	841.73 ± 187.29 (1.2)
+TP-3654	200	10.45 ± 1.95 * (1.5)	388.45 ± 40.35 ** (2.6)
+TP-3654	300	13.19 ± 2.74 (1.2)	261.46 ± 55.67 *** (3.8)
+TP-3654	500	16.46 ± 2.58 (0.9)	153.79 ± 28.65 *** (6.5)
+Ko143	1000	10.28 ± 1.78 * (1.5)	208.89 ± 29.46 *** (4.8)
**Treatment**	**Concentration** **(nM)**	**pcDNA3.1-HEK293 (Parental) (nM)**	**R482-HEK293** **(Resistant) (nM)**
Topotecan	-	24.19 ± 4.95 (1.0)	430.30 ± 67.85 (1.0)
+TP-3654	100	24.72 ± 5.60 (1.0)	149.56 ± 31.21 ** (2.9)
+TP-3654	200	26.34 ± 6.76 (0.9)	81.88 ± 14.13 *** (5.3)
+TP-3654	300	24.80 ± 5.40 (1.0)	73.56 ± 11.35 *** (5.8)
+TP-3654	500	27.06 ± 6.68 (0.9)	49.04 ± 8.91 *** (8.8)
+Ko143	1000	26.34 ± 5.49 (0.9)	41.75 ± 8.49 *** (10.3)
		**(nM)**	**(nM)**
SN-38	-	2.61 ± 0.80 (1.0)	177.87 ± 31.96 (1.0)
+TP-3654	100	2.67 ± 0.48 (1.0)	53.98 ± 17.04 ** (3.3)
+TP-3654	200	2.83 ± 0.55 (0.9)	27.40 ± 7.04 ** (6.5)
+TP-3654	300	2.34 ± 0.50 (1.1)	34.31 ± 5.22 ** (5.2)
+TP-3654	500	2.55 ± 0.55 (1.0)	25.26 ± 4.19 ** (7.0)
+Ko143	1000	2.73 ± 0.76 (1.0)	9.35 ± 1.76 *** (19.0)
		**(nM)**	**(nM)**
Mitoxantrone	-	4.28 ± 0.39 (1.0)	114.49 ± 9.59 (1.0)
+TP-3654	100	3.88 ± 0.29 (1.1)	45.56 ± 5.97 *** (2.5)
+TP-3654	200	4.02 ± 0.33 (1.1)	28.76 ± 4.28 *** (4.0)
+TP-3654	300	3.98 ± 0.38 (1.1)	29.92 ± 5.17 *** (3.8)
+TP-3654	500	4.20 ± 0.36 (1.0)	19.06 ± 3.83 *** (6.0)
+Ko143	1000	4.15 ± 0.48 (1.0)	12.88 ± 1.79 *** (8.9)

Abbreviation: FR, fold-reversal. ^†^ IC_50_ values are mean ± SD calculated from dose–response curves obtained from at least three independent experiments using cytotoxicity assay as described in Section 4. ^‡^ FR values were calculated by dividing IC_50_ values of cells treated with a particular substrate drug by IC_50_ values of cells treated with the same substrate drug in the presence of TP-3654 or Ko143. * *p* < 0.05; ** *p* < 0.01; *** *p* < 0.001.

## Data Availability

The data presented in this study are available on request from the corresponding author.

## Supplementary Materials

The supplementary data are available online at https://www.mdpi.com/article/10.3390/ijms22179440/s1.
