# Supplementary material for: The Second-Generation PIM Kinase Inhibitor TP-3654 Resensitizes ABCG2-Overexpressing Multidrug-Resistant Cancer Cells to Cytotoxic Anticancer Drugs
Source: Int J Mol Sci. 2021 Aug 30;22(17):9440. doi: 10.3390/ijms22179440 (PMC8431370; doi:10.3390/ijms22179440)
Supplement: Supplementary file 1 [file ijms-22-09440-s001.zip › ijms-1319145-supplementary.pdf]

Supplementary Materials

The Second-Generation PIM Kinase Inhibitor TP-3654 Resensitizes ABCG2-Overepressing Multidrug-Resistant Cancer Cells to Cytotoxic Anticancer Drugs

Chung-Pu Wu, Yan-Qing Li, Ya-Chen Chi, Yang-Hui Huang, Tai-Ho Hung, and Yu-Shan Wu

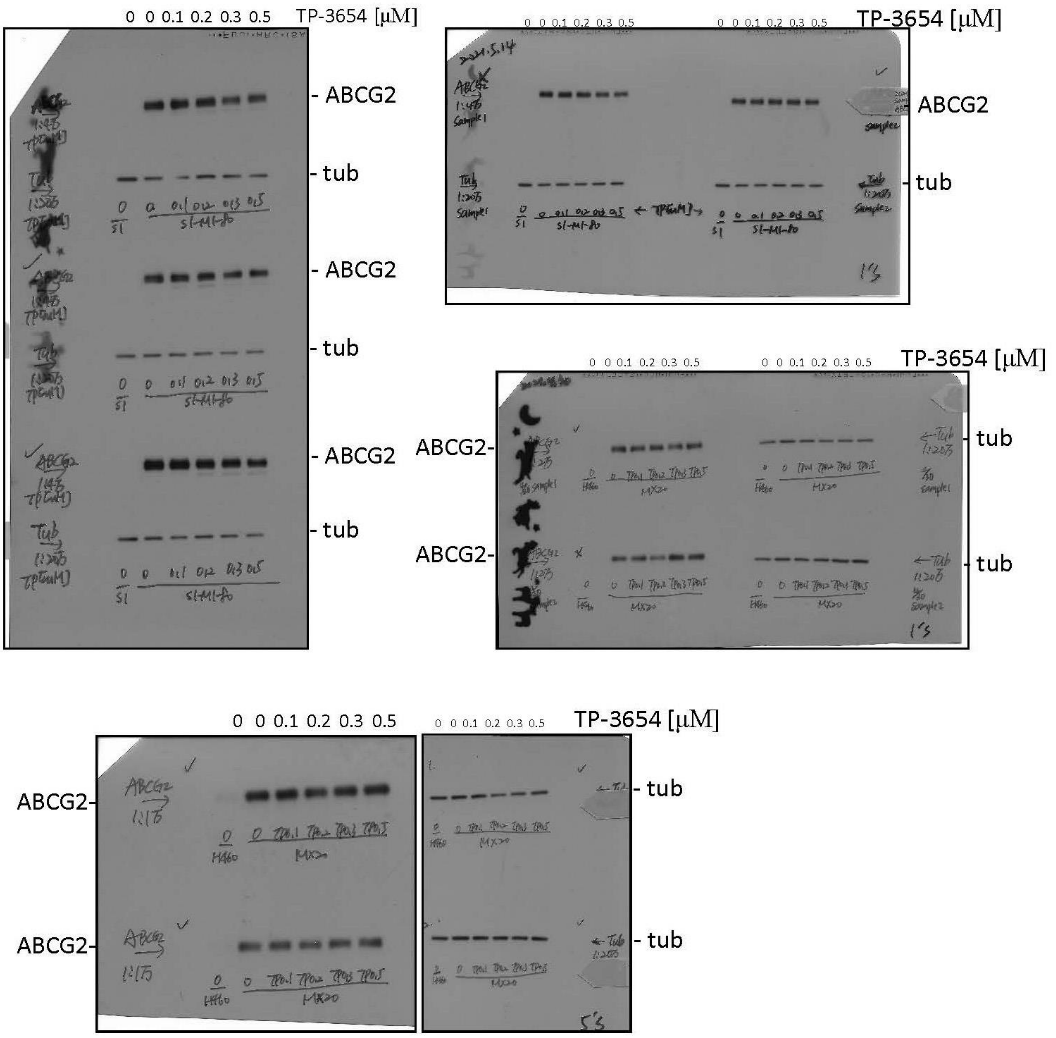

Figure S1: The effect of TP-3654 on the protein expression of ABCG2 in human S1 and S1-M1-80 colon cancer cells, and human H460 and H460-MX20 NSCLC cells.
